# Supplementary material for: Higher temperature accelerates the aging-dependent weakening of the melanization immune response in mosquitoes
Source: PLoS Pathog. 2024 Jan 10;20(1):e1011935. doi: 10.1371/journal.ppat.1011935 (PMC10805325; doi:10.1371/journal.ppat.1011935)
Supplement: S1 Fig — Time course of OD490 measurements of L-DOPA plus water for 30 min. The lower end of the scale is amplified on the right. No meaningful auto-oxidation of L-DOPA was detected. Each circle marks the mean, and whiskers indicate the S.E.M. (PDF) [file ppat.1011935.s001.pdf]

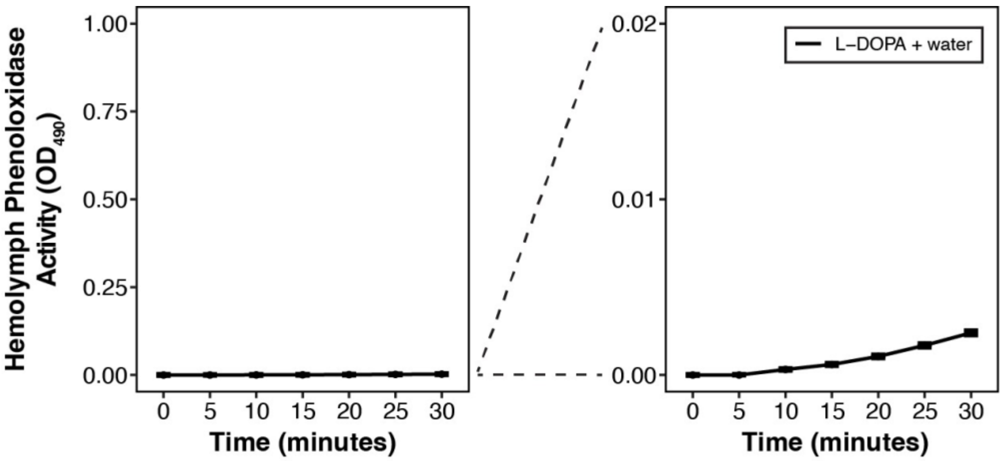

S1 Fig. Auto-oxidation of exogenous L-DOPA is negligible. Time course of OD_{490} measurements of L-DOPA plus water for 30 min. The lower end of the scale is amplified on the right. No meaningful auto-oxidation of L-DOPA was detected. Each circle marks the mean, and whiskers indicate the S.E.M.
