# Supplementary material for: Higher temperature accelerates the aging-dependent weakening of the melanization immune response in mosquitoes
Source: PLoS Pathog. 2024 Jan 10;20(1):e1011935. doi: 10.1371/journal.ppat.1011935 (PMC10805325; doi:10.1371/journal.ppat.1011935)
Supplement: S2 Fig — Time course of OD490 measurements of hemolymph for 30 min. The lower end of the scale is amplified on the right. No meaningful melanization was detected in the absence of exogenous L-DOPA. Each circle marks the mean, and whiskers indicate the S.E.M. (PDF) [file ppat.1011935.s002.pdf]

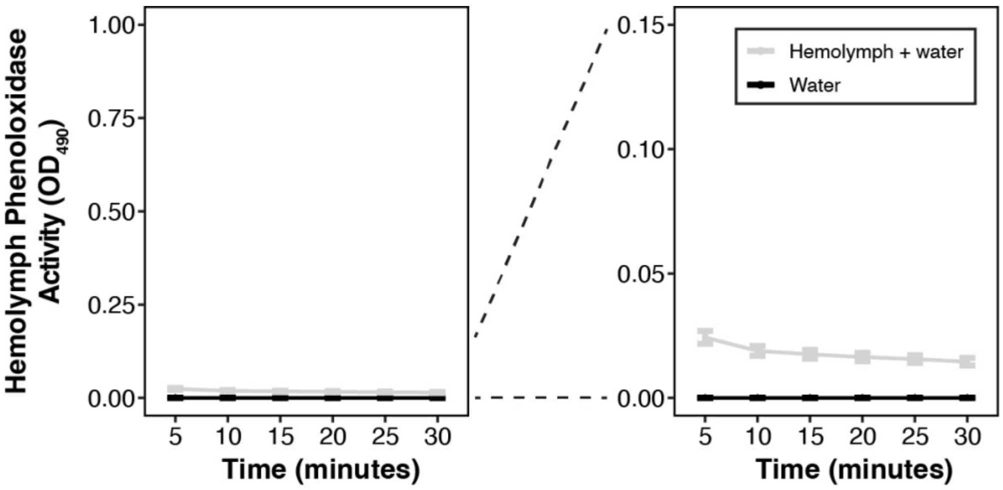

S2 Fig. Melanization in isolated hemolymph is negligible without the addition of the phenoloxidase substrate, L-DOPA. Time course of OD_{490} measurements of hemolymph for 30 min. The lower end of the scale is amplified on the right. No meaningful melanization was detected in the absence of exogenous L-DOPA. Each circle marks the mean, and whiskers indicate the S.E.M.
