# Supplementary material for: Higher temperature accelerates the aging-dependent weakening of the melanization immune response in mosquitoes
Source: PLoS Pathog. 2024 Jan 10;20(1):e1011935. doi: 10.1371/journal.ppat.1011935 (PMC10805325; doi:10.1371/journal.ppat.1011935)
Supplement: S3 Fig — A. Melanization potential, aggregated by temperature and immune treatment, irrespective of age. B. Melanization potential, aggregated by age and immune treatment, irrespective of temperature. C. Melanization potential, aggregated by immune treatment, irrespective of temperature or age. Column height marks the raw mean, and whiskers indicate the S.E.M. The same measurements are plotted in S3 and S4 Figs, but grouped or arranged differently, with aggregated data shown in this figure. The estimated marginal means of these data, resulting from the linear model, are presented in Fig 3. (PDF) [file ppat.1011935.s003.pdf]

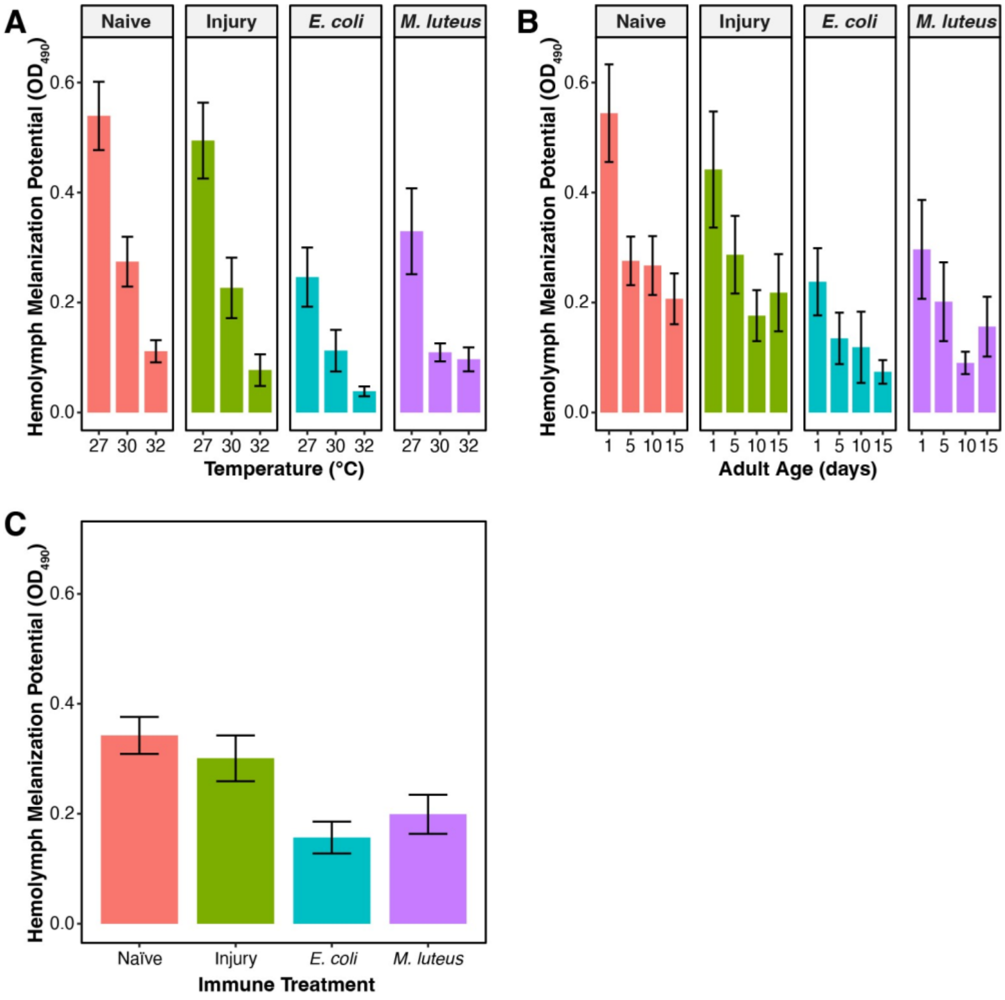

S3 Fig. Raw means of melanization potential, aggregated by temperature, age, and immune treatment. A. Melanization potential, aggregated by temperature and immune treatment, irrespective of age. **B.** Melanization potential, aggregated by age and immune treatment, irrespective of temperature. **C.** Melanization potential, aggregated by immune treatment, irrespective of temperature or age. Column height marks the raw mean, and whiskers indicate the S.E.M. The same measurements are plotted in S3 and S4 Figs, but grouped or arranged differently, with aggregated data shown in this figure. The estimated marginal means of these data, resulting from the linear model, are presented in Fig 3.
