# Supplementary material for: Higher temperature accelerates the aging-dependent weakening of the melanization immune response in mosquitoes
Source: PLoS Pathog. 2024 Jan 10;20(1):e1011935. doi: 10.1371/journal.ppat.1011935 (PMC10805325; doi:10.1371/journal.ppat.1011935)
Supplement: S5 Fig — A. Melanization activity over time, aggregated by temperature and immune treatment, irrespective of age. B. Melanization activity over time, aggregated by age and immune treatment, irrespective of temperature. C. Melanization activity over time, aggregated by immune treatment, irrespective of temperature or age. Each circle marks the raw mean, and whiskers indicate the S.E.M. The same measurements are plotted in S5 and S6 Figs, but grouped or arranged differently, with aggregated data shown this figure. The estimated marginal means of these data, resulting from the linear mixed model, are presented in Fig 5. (PDF) [file ppat.1011935.s005.pdf]

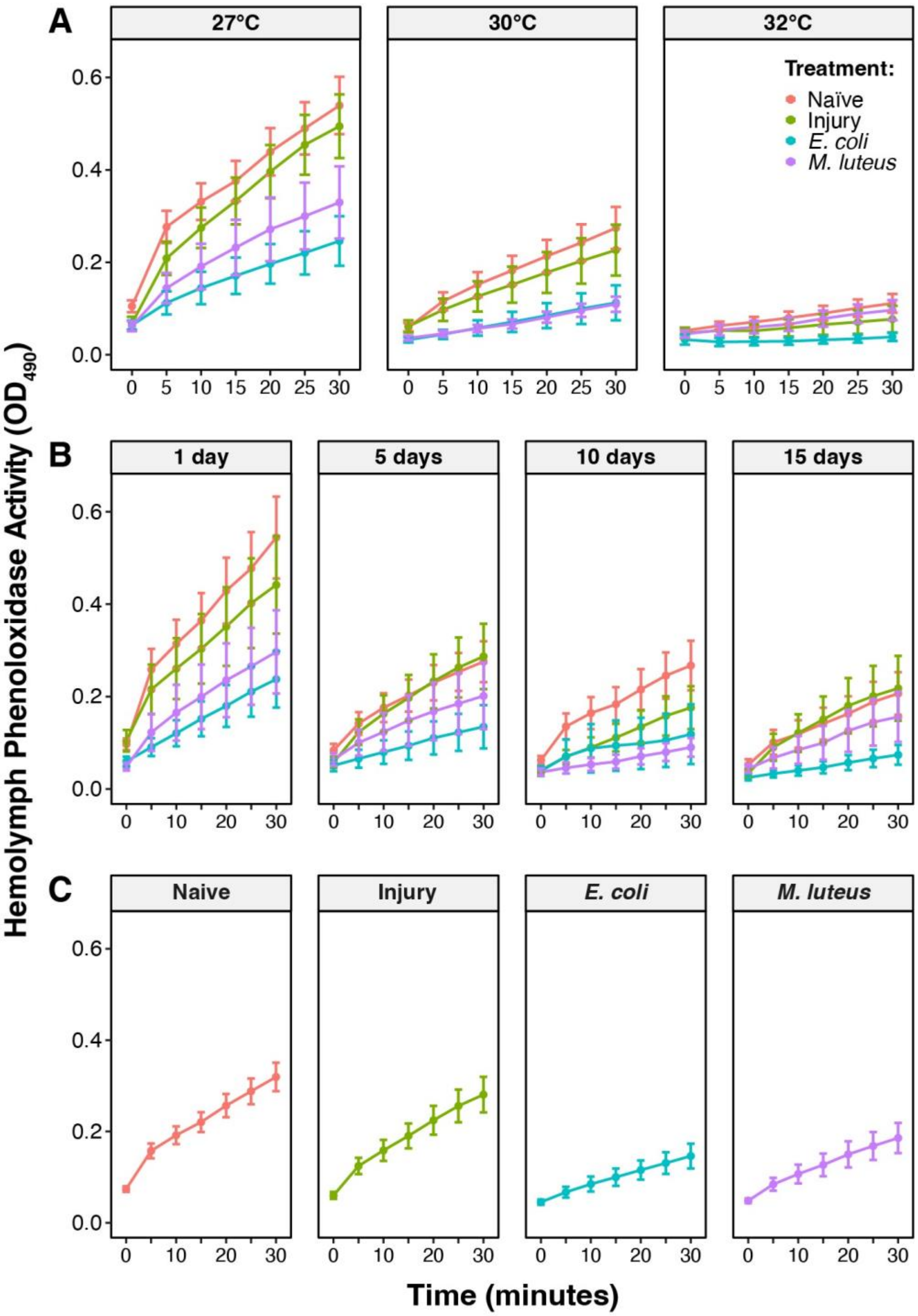

S5 Fig. Raw means of melanization over time, aggregated by temperature, age, and immune treatment. A. Melanization activity over time, aggregated by temperature and immune treatment, irrespective of age. **B.** Melanization activity over time, aggregated by age and

immune treatment, irrespective of temperature. **C.** Melanization activity over time, aggregated by immune treatment, irrespective of temperature or age. Each circle marks the raw mean, and whiskers indicate the S.E.M. The same measurements are plotted in S5 and S6 Figs, but grouped or arranged differently, with aggregated data shown this figure. The estimated marginal means of these data, resulting from the linear mixed model, are presented in Fig 5.
