# Supplementary material for: Higher temperature accelerates the aging-dependent weakening of the melanization immune response in mosquitoes
Source: PLoS Pathog. 2024 Jan 10;20(1):e1011935. doi: 10.1371/journal.ppat.1011935 (PMC10805325; doi:10.1371/journal.ppat.1011935)
Supplement: S8 Fig — Column height marks the raw mean, and whiskers indicate the S.E.M. The same measurements are plotted in S7 and S8 Figs, but grouped or arranged differently, with unaggregated data shown in this figure. The estimated marginal means of these data, resulting from the linear model, are presented in Fig 8. (PDF) [file ppat.1011935.s008.pdf]

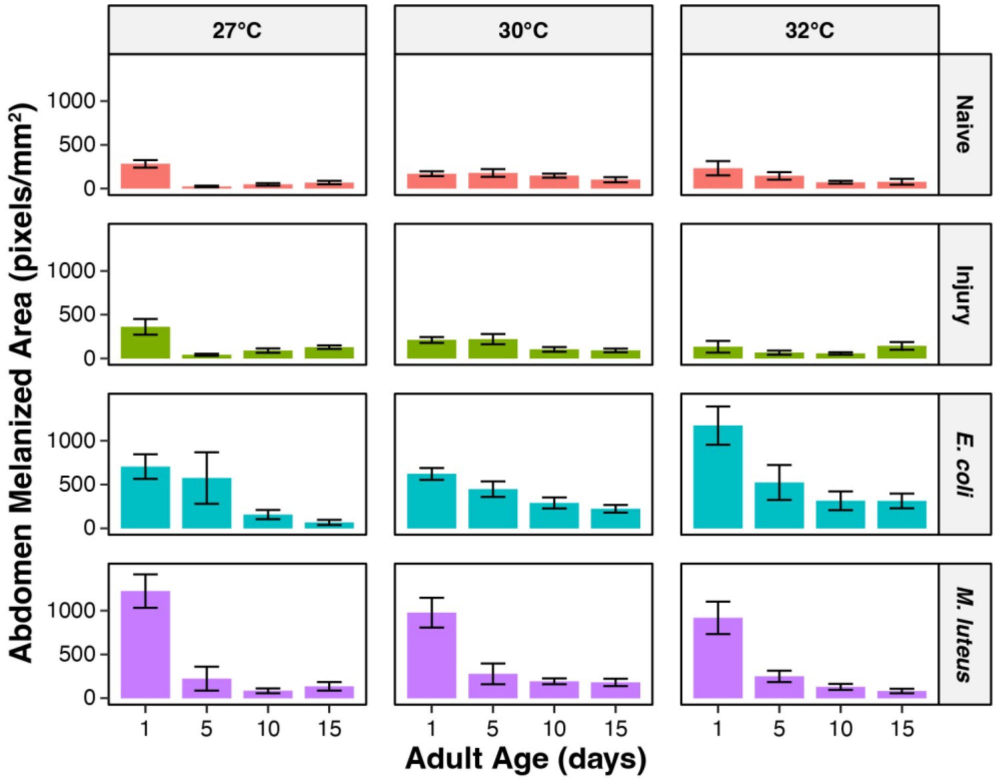

S8 Fig. Raw means of melanin deposition on the dorsal abdominal wall. Column height marks the raw mean, and whiskers indicate the S.E.M. The same measurements are plotted in S7 and S8 Figs, but grouped or arranged differently, with unaggregated data shown in this figure. The estimated marginal means of these data, resulting from the linear model, are presented in Fig 8.
