# Supplementary material for: Higher temperature accelerates the aging-dependent weakening of the melanization immune response in mosquitoes
Source: PLoS Pathog. 2024 Jan 10;20(1):e1011935. doi: 10.1371/journal.ppat.1011935 (PMC10805325; doi:10.1371/journal.ppat.1011935)
Supplement: S9 Fig — A. Melanin deposition, aggregated by temperature and immune treatment, irrespective of age. B. Melanin deposition, aggregated by age and immune treatment, irrespective of temperature. C. Melanin deposition, aggregated by immune treatment, irrespective of temperature or age. Column height marks the mean, and whiskers indicate the S.E.M. The same measurements are plotted in S9 and S10 Figs, but grouped or arranged differently, with aggregated data shown in this figure. The estimated marginal means of these data, resulting from the linear model, are presented in Fig 9. (PDF) [file ppat.1011935.s009.pdf]

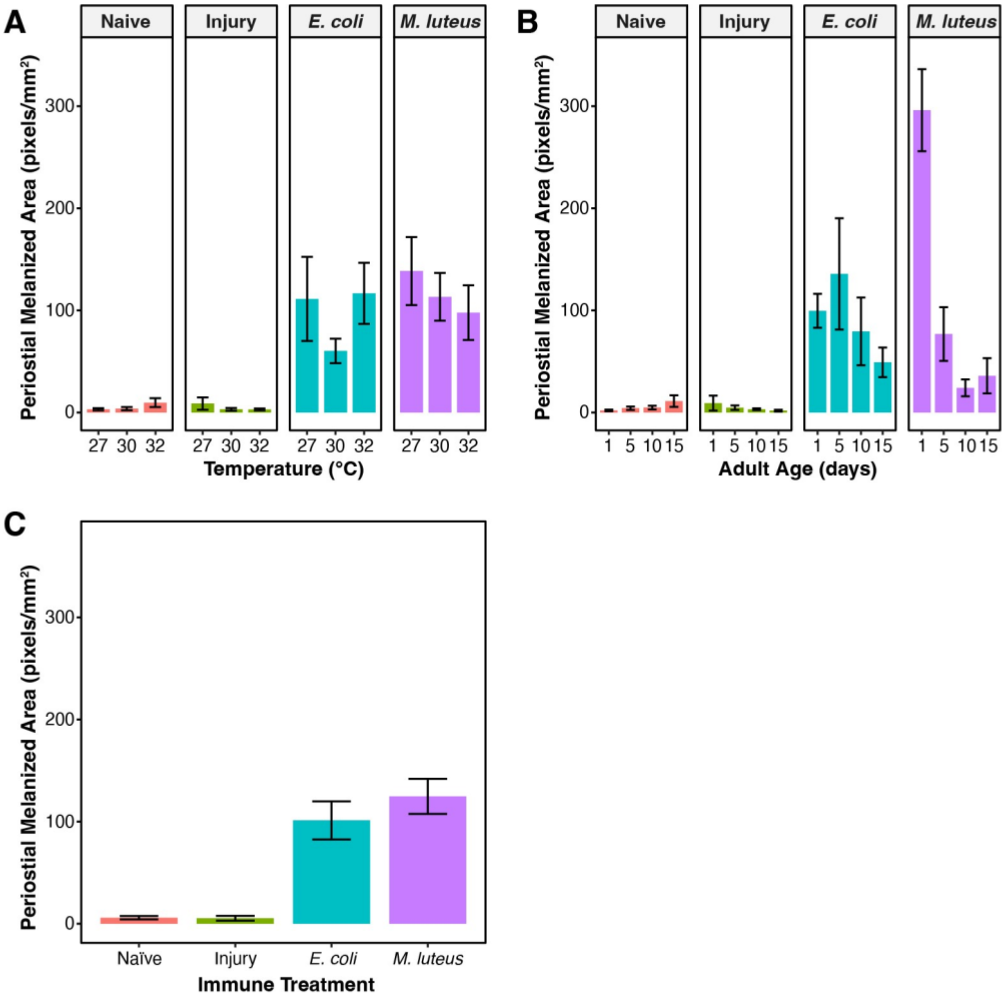

S9 Fig. Raw means of melanin deposition within the periostial regions and posterior excurrent opening, aggregated by temperature, age, and immune treatment. A. Melanin deposition, aggregated by temperature and immune treatment, irrespective of age. **B.** Melanin deposition, aggregated by age and immune treatment, irrespective of temperature. **C.** Melanin deposition, aggregated by immune treatment, irrespective of temperature or age. Column height marks the mean, and whiskers indicate the S.E.M. The same measurements are plotted in S9 and S10 Figs, but grouped or arranged differently, with aggregated data shown in this figure. The estimated marginal means of these data, resulting from the linear model, are presented in Fig 9.
