# Supplementary material for: Higher temperature accelerates the aging-dependent weakening of the melanization immune response in mosquitoes
Source: PLoS Pathog. 2024 Jan 10;20(1):e1011935. doi: 10.1371/journal.ppat.1011935 (PMC10805325; doi:10.1371/journal.ppat.1011935)
Supplement: S10 Fig — Column height marks the mean, and whiskers indicate the S.E.M. The same measurements are shown in S9 and S10 Figs, but grouped or arranged differently, with unaggregated data shown in this figure. The estimated marginal means of these data, resulting from the linear model, are presented in Fig 10. (PDF) [file ppat.1011935.s010.pdf]

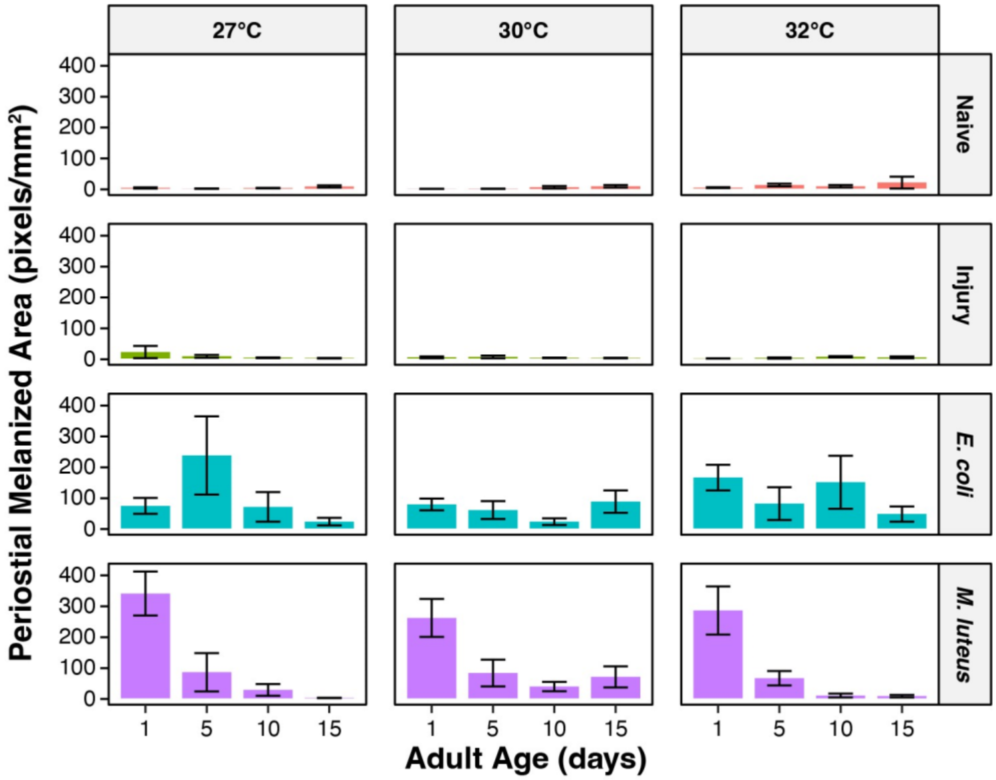

S10 Fig. Raw means of melanin deposition within the periostial regions and posterior excurrent opening. Column height marks the mean, and whiskers indicate the S.E.M. The same measurements are shown in S9 and S10 Figs, but grouped or arranged differently, with unaggregated data shown in this figure. The estimated marginal means of these data, resulting from the linear model, are presented in Fig 10.
