# Supplementary material for: Higher temperature accelerates the aging-dependent weakening of the melanization immune response in mosquitoes
Source: PLoS Pathog. 2024 Jan 10;20(1):e1011935. doi: 10.1371/journal.ppat.1011935 (PMC10805325; doi:10.1371/journal.ppat.1011935)
Supplement: Attachment — Submitted filename: 2023 12 Martin and Hillyer PLOS Pathogens Response.pdf [file ppat.1011935.s014.pdf]

Reviewer Comments (if any, and for reference):

Reviewer's Responses to Questions

Part I - Summary

Please use this section to discuss strengths/weaknesses of study, novelty/significance, general execution and scholarship.

Reviewer #1: This is an interesting paper with important implications for disease resistance/tolerance and transmission in insects – and particularly in vectors. It also has interesting implications overall for host-parasite interactions in relatively short-lived insects and pressures that could shape the evolution of parasite life cycles in mosquitoes. If you develop too quickly as a parasite that needs to migrate through the hemolymph to the salivary glands, perhaps you encounter too strong an immune response; however, the timing of development could be under selective pressure to match the senescence of the immune system. It certainly has me thinking! I have mainly minor suggestions. The general execution is sound and I have really only a few suggestions for interpretation of the data.

Author response: Reviewer 1 is enthusiastic about the manuscript and declares that the findings are thought provoking. We infer that the reviewer sees the high impact of this study. We agree.

Reviewer #2: This manuscript "Warmer temperature accelerates the aging-dependent weakening of the melanization immune response in mosquitoes" explores the interaction of increasing temperature and age on the melanization response. Laboratory strain G3 mosquitoes were reared at 3 different temperatures selected to represent a range expected from global climate change. From these conditions, mosquitoes at 4 different ages were subjected to immune challenge, injury, or control. 24 h post treatment, the melanization response was assayed using an enzymatic assay and by observation of melanin deposition in the cuticle. Declines in melanization were observed with increased age and temperature. Furthermore, temperature speeds up the age-dependent decline. Immune challenge also, reduced melanization enzymatic activity, but increased melanotic deposits. In addition to the abdominal cuticles, melanization of the periostial region was also quantified.

Author response: In this portion of the critique, Reviewer 2 summarizes the manuscript.

Part II – Major Issues: Key Experiments Required for Acceptance

Please use this section to detail the key new experiments or modifications of existing experiments that should be <u>absolutely</u> required to validate study conclusions.

Author preamble: In this section the reviewers make recommendations and other comments, and we have modified the manuscript to incorporate and address these recommendations and comments (details below). We do not see in this section any experiments that the reviewers deem to be "absolutely required to validate study conclusions." In fact, reviewer 1 notes above that "the general execution is sound and I have really only a few suggestions for interpretation

of the data", and reviewer 2 notes below "overall the manuscript is clear and conclusions are generally supported by the data". If we have read this incorrectly, we would appreciate if the editor clarifies what is absolutely required.

<u>Reviewer #1:</u>

I suggest "higher" temperature instead of "warmer" temperature throughout. It's finicky and not that important, but grammatically appropriate.

Author response: We agree with this suggestion. Throughout the manuscript and the supplement, we substituted the word "warmer" with the word "higher".

Thank for the experimental overview – that is very helpful!

Author response: You are welcome. For this type of study, we find that presenting a graphical experimental overview adds clarity.

Did any of the mosquitoes die from infection or injury? I.e. did the infection select for any particular mosquitoes?

Author response: Yes, mosquito survival varied between the experimental groups. We have just completed the experimental portion of a separate study where we tracked mosquito survival as well as infection intensity under these same treatment conditions. This will be a follow-up paper and is another massive study where survival was recorded in over 8000 mosquitoes and infection intensity was recorded in over 600 mosquitoes.

Because of the size and extent of both the present study (melanization potential in nearly 7000 mosquitoes and melanin deposition in nearly 700 mosquitoes, split among 48 treatment combinations) and the follow-up study (see paragraph above), it is impractical to combine both studies into a single manuscript.

But mosquito survival is an important point to recognize even when it does not impact our current analysis or conclusions. Those mosquitoes that survive are the only mosquitoes that represent the population, both in nature and in the lab. The melanization potential of dead mosquitoes is uninformative. So, we added a new paragraph in the discussion (next to last paragraph) that reads: *"This study did not consider mosquito survival as part of the analysis. However, the risk of death increases with aging (36, 41, 42), higher temperature shortens lifespans (7, 9, 14), and infection decreases survival (42, 83). How age, temperature and infection interact to shape mosquito survival is the focus of ongoing experiments in our laboratory, but the rate of mosquito death does not affect our conclusions on melanization. Here, we only assessed melanization in the surviving mosquitoes because these are the only mosquitoes that, under the conditions tested, would be available for pathogen acquisition and transmission."*.

I was wondering if a useful way to show the interaction between age and temperature would be to express active or melanization as a percent of what was available at day 1. It could show the acceleration of decline at 30 C compared to 27 C even more.

Author response: We went ahead and created new graphs that displayed the data as a percent of what was available on day 1 and found, in our view, that the point was not made any clearer than in the original graphics. Therefore, because we already have 11 figures and 10 supplementary figures, we decided not to include them in the revision. However, we felt that including another example in the text would be useful, so below the example using naïve mosquitoes only we now include an example on *M. luteus* infected mosquitoes. In the paragraph that invokes figure 4 (first section of the results), the new example reads, *"As another example using* M. luteus*-infected mosquitoes only, and relative to 1-day-olds maintained at 27°C, 1-day-olds maintained at 32°C had a melanization potential that was 82% lower. As mosquitoes aged from 1 to 10 days old, the melanization potential of mosquitoes at 27°C decreased by 53%, but the melanization potential of mosquitoes at 32°C remained the same"*.

On that note, I'm also curious about what is happening at 32 and perhaps noting that the interaction of aging and temperature seem null at the highest temperature. PO seems to be so low at 32 C to begin with that there really isn't a decline with age at all; and I suspect that a 1-day-old mosquito at 32 C isn't equivalent to 15-day-old mosquito at 30 C (i.e. they both have approx. the same PO response).

Author response: This is a good observation. We believe that the interactive decline with higher temperature occurs during the immature stages. To make this case, we introduced the following paragraph into the discussion (sixth paragraph): *"The aging-dependent weakening of melanization potential is less pronounced when the temperature is higher. This is largely because mosquitoes reared at higher temperatures eclose with a lower melanization potential, which resembles that of older adults that had been reared at cooler temperatures. While this may seem to indicate a lack of aging-based weakening at higher temperatures, it is more likely that the aging-based weakening at higher temperature occurs during the immature stages and prior to adult emergence, reducing the initial adult potential for melanization. This notion is supported by the findings that larvae have greater melanization activity than adults (51), and that higher temperature reduces cuticular melanization (74, 79), which is needed during eclosion (69, 70)."*.

You say on line 412 that heat weakens melanization, but it does seem that in some cases the melanization response is actually fairly robust, if not stronger, at higher temps (e.g. with E. coli infection). It's a conundrum to some extent that PO can be so low at 32 C but there can still be a strong melanization response – do you think they just don't need as much PO at higher temps? I.e. it's hot enough that the enzyme works quickly enough that they don't require as much enzyme. Because they are reared at 30 C, this could represent an acclimation effects rather than an aging effect. It might be helpful to mention this in the discussion.

Author response: Higher temperature strengthening is not seen for melanization potential. Moreover, this phenotype is only seen for melanin deposition after infection with *E. coli*. We believe that this is because the higher temperature is closer to the optimal growth temperature of *E. coli*. To address this, we added the following paragraph to the discussion (eight paragraph): "*Although infection for 24 h decreases melanization potential, it increases melanin deposition. This is because infection activates the melanization response, leading to the phagocytosis and sequestration of melanized bacteria by sessile hemocytes on the abdominal wall (22, 53, 60-62). Interestingly, melanin deposition in mosquitoes infected with* E. coli *was greatest at the highest temperature, even when melanization potential was not. This may at first suggest a beneficial effect of higher temperature, but we do not believe that this is the case. Instead, we hypothesize that because the highest temperature is closer to the optimal growth temperature of* E. coli*, bacterial proliferation increases, leading to a greater melanization response. The optimal temperature for the growth of* M. luteus *is lower than for* E. coli*, and hence, this rising temperature trend is not observed for* M. luteus.".

Reviewer #2:

Overall the manuscript is clear and conclusions are generally supported by the data. Given that the effects of increasing temperature and age have independently been studied by these authors and others in different systems, the combined effect is not unexpected, and without a clear mechanism does not make a major impact for the field

Author response: We thank the reviewer for noting that the manuscript is clear and that the conclusions are supported by the data. However, we unambiguously disagree with the reviewer statement on impact. If we simplify the reviewer's argument, if condition A has effect X and condition B also has effect X, then when both conditions are present the effect must be 2X. This is very often not the case. Our study demonstrates that higher temperature accelerates immune senescence in mosquitoes, and we believe that this is the first demonstration of this interactive effect in any insect. The relevance and impact of this study is high, especially when considering the current trend of warming global temperatures.

The importance of this work is about mosquito vectorial capacity, so examining how age/temp/immunity are affected by blood feeding is crucial. This is acknowledged in the manuscript as something for future work, but should be included in these studies.

Author response: Follow-up experiments in blood-fed mosquitoes will be conducted at a later time. We noted this in the discussion of the original version of the manuscript (second to last paragraph of the original submission). A current goal in our laboratory is to uncover whether temperature and age interact to shape the various components of the immune system. We started with melanization are now conducting experiments on lysis and phagocytosis. We are doing these studies in non-blood fed mosquitoes and will use these findings to then select phenotypes to assess after a blood meal. Melanization is a promising phenotype to assess in a future study, and we note this in the discussion.

There are advantages to beginning these experiments in non-blood fed mosquitoes. For example, we can assay 1-day-old mosquitoes (mosquitoes do not blood feed at this age). Moreover, we believe that these data are applicable to other ectothermic and poikilothermic insects, like agricultural pests and pollinators. Most of these insects are not hematophagous, so we consider that introducing a blood meal narrows how our findings can be broadly interpreted to the Class Insecta.

The lack of mechanism also detracts from the overall impact. It is suggested that for both temperature and age, hemocytes have been shown to decrease in mosquitoes or other insects, so perhaps this is the mechanism for the further decline, but it is not addressed here by hemocyte number or other mechanistic insight. There are studies that have chemically depleted hemocytes that could be employed into these analyses.

Author response: We agree that having a universal mechanism would be ideal, but we reiterate that the interactive effects of temperature and age on immunity have not been studied or quantified in any insect system, so the findings are novel and impactful regardless of the definitive mechanism(s). As for the mechanism, we believe that multiple mechanisms are likely converging to result in the observed phenotypes. In other words, a unifying mechanism is unlikely.

As for chemically depleting hemocytes, we believe that this is a useful tool for the study of insect immunity but is unlikely to uncover a hemocyte-mediated mechanism for the melanization phenotype even if the mechanism was hemocyte mediated. Chemical depletion of hemocytes is done via clodronate liposomes. These liposomes deplete phagocytic hemocytes, and therefore, the granulocyte population. The majority of phenoloxidase is produced by oenocytoids, and these hemocytes are not professional phagocytes. Oenocytoids are most likely only marginally affected by clodronate liposomes.

All that said, in the discussion we already propose mechanisms that could explain the observed phenotypes. In our lab, we have begun experiments testing how temperature and age interact to shape the hemocyte population. These experiments are in their early stages and will take a significant amount of time to complete. They will be the focus of a follow-up manuscript.

Part III – Minor Issues: Editorial and Data Presentation Modifications
Please use this section for editorial suggestions as well as relatively minor modifications of existing data that would enhance clarity.

Reviewer #1: (No Response)

Author response: Reviewer 1 did not identify any minor issues.

Reviewer #2:

It is stated that the melanization potential of the hemolymph is being measured. It is more accurate that the current melanization activity of the hemolymph is what is being measured.

Author response: We believe that what we measure in the spectrophotometric assay is more accurately described as "melanization potential" than "melanization activity". The reason is that the assay extracts hemolymph and adds substrate to measure melanization activity, but this melanization activity was not manifesting itself inside the mosquito. If the melanization activity had been already manifesting itself in the mosquito, then we would see melanin deposits in the abdominal wall of all mosquitoes. Moreover, our control hemolymph samples (hemolymph without added L-DOPA) would have been brown instead of clear. This was not the case. We clarify this in the first paragraph of the results section with the following sentence: *"This assay captures melanization potential because it measures the ability of hemolymph to melanize in the event that the phenoloxidase-based cascade is turned on to its fullest"*.

It would be important to challenge the age/temp ranges with bacteria acutely and then performing a melanization assay. This type of assay would more accurately reflect the potential of the hemolymph to mount a melanization response.

Author response: We are not 100% sure what the reviewer means here, but we believe that the recommendation is to infect and immediately measure the melanization potential. If this is the recommendation, then we are certain that the outcome of the spectrophotometric assay would be the same as for uninfected mosquitoes. The reason is that there would not have been time for the transcription and translation of additional enzymes. Also, activation of the system by hemolymph collection would be the same, and saturation with substrate would also be equal. But this is a good point to address, and we clarify our position in the end of the seventh paragraph of the discussion with a new sentence that reads, *"Regardless of the specific reason for the reduction in melanization potential at 24 h following infection, the melanization potential in naïve mosquitoes represents the melanization activity that can take place at the initiation of any infection. The melanization potential in mosquitoes infected for 24 h then informs on how the progression of an infection affects this immune response"*.

It is not reported how many mosquitoes died following immune challenge. The assay might not accurately represent the population if the highly responding individuals die.

Author response: The reviewer is correct that this study does not address mosquito survival. This is the topic of a follow-up study, and we present a more comprehensive explanation for this comment in the "major issues" section for reviewer 1. Moreover, we address this in a new paragraph in the discussion (second to last paragraph). In summary, mosquito survival does not impact our current analysis or conclusions. Those mosquitoes that survive are the only mosquitoes that represent the population, both in nature and in the lab. The melanization potential of dead mosquitoes is uninformative.

There are also overlapping rationales for the declines observed in the enzymatic assay. For age, senescence, presumably hemocyte decline, is cited, which is possibly accelerated with

temperature. With immune challenge, the decrease is enhanced due to the consumption of the melanization components as the enzymatic assays are performed 24 h after the immune challenge. Melanotic deposits in the immune challenged mosquitoes support the immune challenge depletion model, but since both inhibition via senescence or inhibition via consumption have the same effect on the assay, the interpretation is confusing.

Author response: This is a three-variable study with forty-eight different treatment combinations. A single mechanism driving the phenotypes under every condition is highly unlikely, so throughout the manuscript we present and discuss the most likely candidates. We believe that multiple mechanisms converge, and in the laboratory we are currently conducting assays on other immune processes to better understand how temperature and age interact to decouple chronological and physiological age. We are hopeful that, collectively, the present study and the follow-up studies will provide clarity on how temperature alters immune senescence in insects.

Additional changes to the manuscript

1. We modified figure 11. The original figure contained asterisks that were defined in the figure legend. In the revised figure, we include the "asterisk information" in the figure itself instead of using the asterisks. This makes the figure completely understandable without having to read the legend.
2. We made single word changes (additions and deletions) in the several sentences to improve reading clarity.
